# Factors Associated With the Level of Trust in Health Information Robots Among the General Population From a Socioecological Model Perspective: Network Analysis

**DOI:** 10.2196/68299

**Published:** 2025-06-13

**Authors:** Jiukai Zhao, Yuqi Yang, Juanxia Miao, Xue Wang, Dianjun Qi, Shuang Zang

**Affiliations:** 1 Department of Community Nursing School of Nursing China Medical University Shenyang China; 2 School of Nursing Henan University of Science and Technology Luoyang China; 3 Department of General Practice The First Affiliated Hospital of China Medical University Shenyang China

**Keywords:** robots, health information, socioecological model, network analysis, artificial intelligence

## Abstract

**Background:**

Although robots have emerged as a new means of delivering health information, with the advancement of artificial intelligence technology, individuals still face challenges in deciding whether to trust the health information provided by these robots owing to various trust-related factors.

**Objective:**

This study aimed to investigate the factors associated with the level of trust in health information robots among the general population in China from a socioecological model perspective and identify the central indicators based on network analysis.

**Methods:**

A nationwide survey in China was conducted from June 20, 2023, to August 31, 2023, involving 30,054 participants. The level of trust in health information robots was measured using a self-developed questionnaire. Univariate and multivariate generalized linear model analyses were conducted to investigate the factors associated with the level of trust in health information robots. Network analyses were conducted to examine the network structure of trust levels in health information robots and the associated factors.

**Results:**

The results of the multivariate generalized linear model analysis revealed that participants who were diagnosed with chronic diseases; exhibited personality traits of higher agreeableness and openness; had an education level of junior college or higher; reported higher self-rated health status, health literacy, anxiety symptoms, family health, number of house properties, average monthly household income, and perceived social support; and had higher medical insurance coverage showed a positive association with the level of trust in health information robots compared to individuals without these characteristics. However, compared to individuals without these characteristics, being older, having the personality trait of neuroticism, and living in an urban area were negatively associated with the level of trust in health information robots. In addition, using a network approach, central indicators were identified in the network of the level of trust in health information robots and its associated factors, including family health and perceived social support. Finally, agreeableness and educational level appeared upstream of the entire directed acyclic graph, directly influencing the level of trust in health information robots.

**Conclusions:**

Our findings offer a novel perspective on the association between health information robots and trust and contribute to the application and development of artificial intelligence IT. Individuals’ acceptance of and adherence to health information may be enhanced if the factors associated with the level of trust in health information robots are considered.

## Introduction

### Background

Previous studies have demonstrated that nearly half of Chinese patients seek information about their medical conditions from various sources before seeking professional consultation, and this informational need is persistently increasing [1]. Regardless of whether they are patients or caregivers, distinct information needs are among the most prevalent yet unmet needs throughout individuals’ life experiences [2-4]. However, with the advancement of the information age, avenues for accessing information have gone beyond traditional channels, encompassing internet search engines, consulting friends and family, and watching health programs on television [5]. Progress in artificial intelligence (AI) has propelled the application of robots in medical fields [6] such as telemedicine [7], nursing [8], psychological therapy [9], and social assistance [10].

Robots can act as counterparts or mentors. They can effectively enhance the cognitive and emotional development of users [11]. Notably, the capabilities and communication strategies of robots differ from those of traditional computing devices because robots can interact with users through facial expressions, body movements, and natural language [5]. Consequently, robots can be programmed using AI technologies to provide patients with tailored diagnostic and therapeutic information, thereby meeting individual demands for health services [12]. For example, one study found that, compared to traditional video health education, communication with natural language robots that exhibit facial expressions significantly improved older adults’ learning motivation, health knowledge, and health literacy [5].

China has emerged as the largest global market for robotics applications [13]. The application of robots for information provision in China is currently focused on older adult care and is expected to expand to broader and more accessible contexts in the future [14]. For example, many manufacturers in China are currently working on optimizing the interactions between users and health information chatbots to attract more users [15]. However, despite the emergence of robots as a novel avenue for delivering health information, trust, a critical element in the use of information and fulfillment of information needs, is still lacking [16]. For instance, research has indicated that individuals who trust digital health information are more inclined to engage in various online health-related activities that cater to their emotional and informational needs [17].

Determining whether to place trust in the health information supplied by robots can be a multifaceted choice because previous encounters with the information source and medium, along with the attributes of the source itself, may influence user judgment [18]. Trust is a psychological condition that involves the willingness to be vulnerable, arising from positive expectations regarding others’ intentions or behavior within the context of risk and mutual dependence [19]. For example, one study clarified that trust is a critical factor in any human-robot relationship because it directly influences individuals’ willingness to accept the information generated by robots and adhere to their recommendations [20]. Another systematic review identified trust as a key factor influencing individual attitudes and beliefs regarding social robots [21]. Therefore, it is essential to study the factors associated with the level of trust in health information robots.

According to the technology acceptance model (TAM), an individual’s willingness to accept new technology is primarily influenced by 4 factors: perceived usefulness, perceived ease of use, behavioral intention, and actual use [22]. Within this theoretical framework, trust is regarded as a crucial component of behavioral intention. Specifically, individuals are more likely to evaluate the accuracy, potential misinformation, inconsistencies, or need for verification of the health information provided by robots only when they intend to engage with such technology. Therefore, enhancing user trust in the health information provided by robots is essential for increasing behavioral intentions and facilitating the effective implementation of robotic technologies in health services.

In total, 2 systematic reviews have categorized the factors influencing individuals’ trust in health information into 4 domains: individual, interpersonal, societal, and relational [19,23]. Other studies involving 400 and 388 Chinese patients used an extended TAM with trust and found that personal behavior and privacy significantly influenced patients’ acceptance of medical service robots [24,25]. However, previous studies have only addressed the association between the information provided by robots and trust through a few isolated factors, thus lacking a comprehensive analysis of the factors associated with trust. Therefore, we used the socioecological model as a comprehensive framework to explore the factors associated with the level of trust in health information robots to the general Chinese population.

The socioecological model comprises 5 interconnected levels: individual characteristics, individual behaviors, interpersonal networks, community factors, and policy [26]. These levels work synergistically to provide a comprehensive framework for understanding health-related phenomena and factors associated with personal behavioral motivation from different perspectives [27]. For example, one study used a socioecological model to explore the factors associated with self-care in patients with heart failure and found that this model provided a better understanding of the potential processes and relationships among these factors [28]. Another study used a socioecological model to examine factors related to the intention of early career researchers to share their research findings with the academic community [29]. Therefore, this study used a socioecological model to gain a better understanding of individuals’ trust in health information robots from different dimensions.

However, relying solely on traditional regression methods cannot accurately analyze the key factors associated with the level of trust in health information robots. Therefore, we applied a network analysis to explore the complex interrelationships among these factors in the model. Network analysis is a sophisticated methodology that delineates the intricate interactions among study variables by representing them as nodes within an interconnected network, effectively bypassing the limitations of traditional research methods [30]. In addition, it incorporates the use of centrality indicators, which are crucial for investigating the centrality and interconnectedness of psychological traits within a network [30]. Network analysis enables a thorough investigation of the complex interrelations among the elements of the socioecological model as well as their respective significance concerning the level of trust in health information robots.

### Objectives

Therefore, it can be stated that, if individuals trust the health information provided by robots, it will help meet their information needs in the era of AI. To date, no study has analyzed the factors associated with the level of trust in health information robots among the general population in China from a socioecological model perspective. This study aimed to do so. In addition, by using network analysis, another goal of our study was to determine the crucial factors affecting trust in health information robots to the general population. Our study can assist policy makers and public health planners in devising more effective health promotion strategies in the era of AI while also aiding in the design of more efficient health information dissemination tactics that are better suited to meet people’s needs, ultimately enhancing public health.

## Methods

### Study Design and Participants

Data were obtained from the Psychology and Behavior Investigation of Chinese Residents database, and a nationwide survey was conducted between June 20, 2023, and August 31, 2023. Data collection involved a multistage sampling method comprising 3 phases. First, the study encompassed 33 provincial-level administrative divisions across China (97.06% of the total), including 22 (67%) provinces, 5 (15%) autonomous regions, 4 (12%) municipalities, and 2 (6%) special administrative regions. The cities selected for the survey were based on the population size and economic development of each province or autonomous region to ensure that the distribution of cities within each provincial jurisdiction was accurately represented. Using a random number table, cities numbered 2 to 12 were randomly selected, resulting in a total of 150 cities for the study. Second, among the 150 cities selected, the number of communities to be sampled was determined based on the population size of the administrative districts in which the cities were located. Consequently, 800 communities were selected, maintaining an urban-to-rural area ratio of 3:2, with each city contributing 10 to 60 communities. Third, for residents in each selected community, quota sampling was used to ensure that the sex and age distributions of the participants were closely aligned with the Chinese Seventh National Population Census [31]. The details of the investigation and sampling processes have been outlined in a previous study [32].

The survey was conducted via one-on-one interviews using an electronic questionnaire that was disseminated and coordinated through the Questionnaire Star platform [33], a widely recognized professional web-based survey tool in China. Before distributing the questionnaires, we enlisted data researchers from diverse regions in China and provided them with standardized training. Following this, data were collected. The inclusion criteria for the study participants were as follows: participants must be aged ≥18 years; be of Chinese nationality refers specifically to individuals who hold Chinese citizenship (including having a permanent residence in China with an annual absence not exceeding 1 month); have the capacity to comprehend the questionnaire content; and be capable of completing the questionnaire either independently or with limited assistance from an investigator, ensuring that such assistance did not affect the authenticity of the participants’ responses. The exclusion criteria for study participants were as follows: participants with impaired consciousness, those exhibiting psychiatric disorders, those diagnosed with cognitive impairments, and those who were currently participating in other similar studies or had previously participated in surveys conducted by the Psychology and Behavior Investigation of Chinese Residents. Therefore, the final study cohort comprised 30,054 participants.

### Ethical Considerations

This study was conducted in compliance with the ethical guidelines of the Declaration of Helsinki and was approved by the ethics research committee of the Shandong Provincial Hospital (approval 2023-198). All data in this research do not involve participant compensation. All participants provided written informed consent before taking part. The research protocol was designed to ensure the protection of participants’ privacy and the confidentiality of the collected data.

### Survey Instruments

#### Overview

On the basis of the socioecological model and previous studies [27,34], factors at the individual characteristic level included age, sex, educational level, diagnosis of chronic disease, personality traits, health-related quality of life, and concerns regarding privacy breaches in AI health-monitoring devices. Factors at the individual behavioral level included depression symptoms, anxiety symptoms, self-rated health status, health literacy, and eHealth literacy. The interpersonal network factors encompassed marital status and family health. Factors at the community level included career status, urban-rural distribution, number of house properties, average monthly household income, and previous social support. Finally, the policy level included the type of medical insurance. Considering China’s unique national context, the ranking of medical insurance types was based on the proportion of out-of-pocket expenses after insurance payment, presented in descending order.

**Figure 1:**
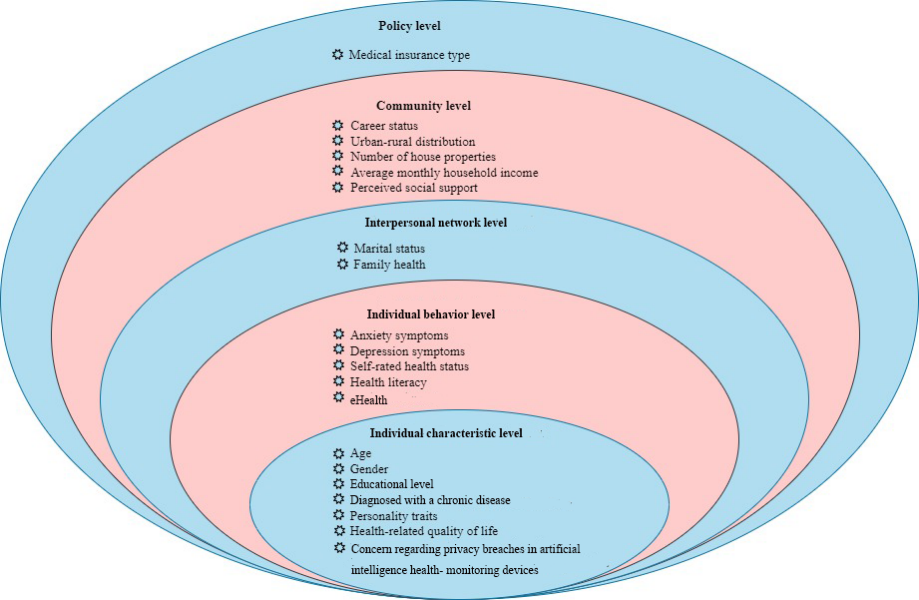
Factors associated with the level of trust in health information robots based on the socioecological model.

Accordingly, this study used a combination of self-developed and standardized questionnaires to examine the factors associated with the level of trust in health information robots based on a socioecological model (Figure 1).

#### Self-Developed Questionnaires

The self-developed questionnaires used in this study included items that assessed participants’ baseline characteristics. These characteristics included demographic factors such as age, sex, education level, marital status, career status, urban-rural distribution, and medical insurance type. In addition, the questionnaires assessed self-rated health status on a scale from 0 to 100, where 0 indicated “the least healthy” and 100 indicated “the healthiest.” Furthermore, the questionnaires evaluated the concern regarding privacy breaches in AI health-monitoring devices, also rated on a scale from 0 to 100 where 0 indicated “no concern” and 100 indicated “concern.” To enhance the participants’ understanding of the term “artificial intelligence health monitoring devices,” this study included the following explanation beneath the question: “Artificial intelligence health monitoring devices refer to systems that employ non-invasive sensors to collect individual data. These devices use AI to compare collected data with large datasets, extract key factors, and conduct intelligent machine learning, thereby enabling real-time assessments of personalized health risk alerts and predictions of health outcomes.” Basic family information was also collected, including the average monthly household income and the number of house properties. Finally, as the dependent variable of this study, the level of trust in health information robots was measured using a self-developed questionnaire, introduced using the following question: “What is your level of trust in AI question-and-answer robots providing health information?” Responses were rated on a scale from 0 to 100, where 0 indicated “distrust” and 100 indicated “trust.”

#### Standardized Questionnaires

##### Personality Traits

Participants’ personality traits were evaluated using the Big Five Inventory–10 (BFI-10), which includes 5 dimensions: extraversion, agreeableness, conscientiousness, neuroticism, and openness [35]. Each item is assessed on a 5-point Likert scale, where 1 indicates “totally disagree” and 5 indicates “totally agree,” including 5 items that are reverse scored. Higher scores indicate a greater presence of each personality trait. The BFI-10 demonstrated high internal consistency (Cronbach α=0.999) in this study.

##### Health-Related Quality of Life

Participants’ health-related quality of life was examined using the Chinese version of the EQ-5D-5L, which includes 5 dimensions: mobility, self-care, performance of usual activities, experience of pain or discomfort, and presence of anxiety or depression [36]. Each item is assessed on a 5-point Likert scale, where 1 indicates “no problems” and 5 indicates “extreme problems.” In addition, the Chinese version of the Health Utilities Index system transforms 5 dimensions of the EQ-5D-5L into a health utility index ranging from −0.391 to 1.000 [37]. An upper limit of 1.000 represents an optimal state of health, whereas a negative score indicates a health status considered worse than death [37]. The EQ-5D-5L demonstrated high internal consistency (Cronbach α=0.730) in this study.

##### Depression Symptoms

Participants’ depression symptoms were examined using the Patient Health Questionnaire–9 (PHQ-9) [38]. Each item is assessed on a 4-point Likert scale, where 0 indicates “never” and 3 indicates “nearly every day.” The PHQ-9 total score ranges from 0 to 27, with higher scores indicating greater severity of depressive symptoms. On the basis of previous studies [39,40], depressive symptoms were defined by a PHQ-9 score of ≥10. The PHQ-9 demonstrated high internal consistency (Cronbach α=0.927) in this study.

##### Anxiety Symptoms

Anxiety symptoms were examined using the Generalized Anxiety Disorder–7 (GAD-7) scale [41]. Each item is assessed on a 4-point Likert scale, where 0 indicates “never” and 3 indicates “nearly every day.” The GAD-7 total score ranges from 0 to 21, with higher scores indicating greater severity of anxiety symptoms. On the basis of previous studies [42,43], anxiety symptoms were operationally characterized using a GAD-7 score of ≥10. The GAD-7 demonstrated high internal consistency (Cronbach α=0.941) in this study.

##### Health Literacy

Participants’ health literacy was examined using the Health Literacy Scale–Short Form (HLS-SF-4) [44]. Each item is assessed on a 4-point Likert scale, where 1 indicates “very difficult” and 4 indicates “very easy.” The HLS-SF-4 total score ranges from 4 to 16, with higher scores reflecting increased levels of health literacy. The HLS-SF-4 demonstrated high internal consistency (Cronbach α=0.883) in this study.

##### eHealth Literacy

Participants’ eHealth literacy was examined using the eHealth Literacy Scale–Short Form (eHEALS-SF) [45]. Each item is assessed on a 5-point Likert scale, where 1 indicates “strongly disagree” and 5 indicates “strongly agree.” The total eHEALS-SF score ranges from 10 to 50, with higher scores reflecting increased levels of eHealth literacy. The eHEALS-SF demonstrated high internal consistency (Cronbach α=0.952) in this study.

##### Family Health

Participants’ family health was examined using the Family Health Scale–Short Form (FHS-SF) [46]. Each item is assessed on a 5-point Likert scale, where 1 indicates strongly disagree “strongly disagree” and 5 indicates “strongly agree.” A total of 3 items were reverse scored. The total FHS-SF score ranges from 10 to 50, with higher scores reflecting increased levels of family health. The FHS-SF demonstrated high internal consistency (Cronbach α=0.807) in this study.

##### Social Support

Participants’ social support was examined using the Perceived Social Support Scale (PSSS) [47]. Each item is assessed on a 7-point Likert scale, with 1 indicating “strongly disagree” and 7 indicating “strongly agree.” The PSSS total score ranges from 3 to 21, with higher scores reflecting greater social support. The PSSS demonstrated high internal consistency (Cronbach α=0.879) in this study.

### Statistical Analyses

#### Overview

First, the Kolmogorov-Smirnov test was used to assess whether the continuous variables followed a normal distribution. A visual check of the *Q*-*Q* plots revealed that these variables did not follow a normal distribution. Descriptive statistics for continuous variables were presented as medians and IQRs, whereas categorical variables were presented as counts and percentages. Second, we investigated the possibility of multicollinearity by determining the variance inflation factor. The analysis indicated no multicollinearity among the independent variables, with a maximum variance inflation factor of 2.942. In addition, a univariate generalized linear model was used to explore the association between the study variables and level of trust in health information robots. Subsequently, variables from the univariate generalized linear model that showed statistical significance (*P*<.05) were incorporated into the multivariate generalized linear model for further analysis. Finally, the network model, which included variables that were statistically linked to the level of trust in health information robots as identified in the multivariate generalized linear model, was estimated using the R packages *bootnet* and *qgraph* (versions 1.4.3 and 1.6.9, respectively; R Foundation for Statistical Computing) [48,49]. Network analysis comprised 2 primary components: a graphical least absolute shrinkage and selection operator (LASSO) network and a directed acyclic graph.

#### Graphical LASSO Network

This network was constructed using Gaussian graphical models [49]. In Gaussian graphical models, the network structure pertains to the arrangement of nodes and edges, providing both visual and mathematical representations of the interconnections between variables. Nodes represent individual variables, including the level of trust in health information robots and its associated factors, whereas edges represent the relationships or interactions between these variables. Within the network layout, the thickness of the edges indicates the strength of the relationships, with blue and red edges signifying positive and negative associations, respectively.

The connections between each pair of nodes were established through partial correlation analysis using Spearman correlation analysis [50,51]. The LASSO was used to prune the network by setting weak correlations to 0, thereby reducing the overall number of connections and removing those with minimal influence [52]. This method ensures that only the nodes with essential connections are retained in the network. Moreover, the hyperparameter for the extended Bayesian information criterion (BIC) was established as 0.5, which achieved an optimal balance between specificity and sensitivity in the extraction of real edges [53]. Network estimation and visualization were conducted using the R packages *bootnet* and *qgraph* [48].

In the current network, the expected influence index of each node was assessed using the R package *qgraph* [48]. The expected influence index is defined as the cumulative value of all the edges connected to an individual node. A higher expected influence index reflects a greater significance of the nodes within the network. Furthermore, the predictability was assessed to determine the extent of a node’s connectivity and its influence on its neighboring nodes [54]. Network visualization used the size of the rings encircling each node to represent the predictability value, which was calculated by applying the prediction function from the *mgm* R package (version 1.2-11) [55].

Network stability was assessed using the case-dropping bootstrap method [51]. This approach entails the systematic removal of an escalating percentage of the dataset, after which the centrality measures are recomputed. A network is considered stable when the omission of a substantial portion of the data does not lead to substantial changes in the indexes [49]. The stability metric is quantified using the correlation stability coefficient (CS-C), which signifies the maximum number of cases that can be eliminated from the dataset without the correlation falling below *r*=0.7 between the centrality indexes of the reduced and original datasets. Typically, a CS-C value of >0.25 is deemed acceptable, but a value of >0.5 is more desirable [49].

A nonparametric bootstrap method was used to assess the stability of edge weights by establishing the 95% CIs. The precision of the 95% CIs indicates the reliability of the edges, with narrower intervals implying a more dependable network structure [51]. Bootstrapping analyses were conducted targeting 95% CIs to scrutinize the variability in the edge or node expected influence across various entities. The strength of the edges or the expected influence of the nodes was deemed statistically significant when 0 was absent from the CIs. Network stability analysis was conducted using the R package *bootnet* [49].

#### Directed Acyclic Graph

A directed acyclic graph can capture the conditional independent associations among nodes in cross-sectional data and identify valid causal associations among them [56]. Therefore, a directed acyclic graph serves as a directed network that indicates the likely direction of the probabilistic relationships between nodes [57]. The Bayesian hill-climbing algorithm and the *bnlearn* package in R were used to compute a directed acyclic graph of the level of trust in health information robots and its associated factors [58]. The algorithm iteratively modifies the edges by adding, deleting, and inverting their direction until the best model fit is reached in accordance with the BIC [59]. This encompasses a repetitive procedure of randomly restarting this approach with diverse edges connecting different pairs of nodes, altering the structure, and implementing 50 separate random restarts to evade the local maxima. On the basis of previous studies, we implemented 100 perturbations for each restart iteration [60-62].

To guarantee the stability of the directed acyclic graph, we used the bootstrap method (1000 bootstrap datasets generated with replacement) to obtain the final structure of the directed acyclic graph, which consists of a 2-step approach [63,64]. First, we assessed the frequency of each edge occurrence in 1000 bootstrapped directed acyclic graphs. We then applied the optimal cutoff point method for edge retention, which resulted in a directed acyclic graph with high specificity and sensitivity. Second, when evaluating the orientation of each edge, if edges aligned in the same direction were present in ≥51% of the 1000 bootstrapped directed acyclic graphs, these directional edges were incorporated into the final directed acyclic graph.

To facilitate the interpretation of the directed acyclic graph, 2 visual depictions of the resultant outputs were developed following the guidance of previous studies [60,61,65]. In the first visual depiction, the thickness of the edges denotes the variation in the BIC values resulting from the removal of the edge from the directed acyclic graph. In the second visual depiction, the thickness of the edges denotes the directional probabilities.

All statistical tests were conducted using a 2-tailed approach, and the threshold for significance was set at *P*<.05. Statistical analyses were conducted using SPSS (version 22.0; IBM Corp) and R (version 4.3.0). A study framework was developed to further clarify the relationships between the different methods and their underlying logic (Figure S1 in Multimedia Appendix 1).

## Results

### Descriptive Statistics

The baseline characteristics of the participants are presented in Table 1, along with a median score of 60 (IQR 44.00-77.00) points for their level of trust in health information robots. Of the 30,054 participants, 15,043 (50.05%) were women, 20,735 (68.99%) lived in urban areas, and 10,614 (35.32%) had an educational level of a bachelor’s degree or higher.

**Table 1:** Baseline characteristics of the participants based on a socioecological model (N=30,054)^a^.

Variable				Values
**Individual characteristic level**				
	Age (y), median (IQR)			43.00 (29.00-54.00)
	**Sex, n (%)**			
		Female	15,043 (50.05)	
		Male	15,011 (49.95)	
	**Educational level, n (%)**			
		Primary school and lower	9243 (30.75)	
		Junior high school and senior school	10,197 (33.93)	
		Junior college and higher	10,614 (35.32)	
	**Diagnosed with a chronic disease, n (%)**			
		No	20,460 (68.08)	
		Yes	9594 (31.92)	
	**Personality traits (score of 1-10), median (IQR)**			
		Extraversion	6.00 (5.00-7.00)	
		Agreeableness	7.00 (6.00-8.00)	
		Conscientiousness	7.00 (6.00-8.00)	
		Neuroticism	6.00 (6.00-7.00)	
		Openness	6.00 (5.00-7.00)	
	Health-related quality of life (score of 1-5), median (IQR)			1.00 (0.94-1.00)
	Concern regarding privacy breaches in artificial intelligence health-monitoring devices (score of 0-100), median (IQR)			64.00 (45.00-82.00)
**Individual behavior level**				
	**Depression symptoms, n (%)**			
		Absence	24,189 (80.49)	
		Presence	5865 (19.51)	
	**Anxiety symptoms, n (%)**			
		Absence	26,228 (87.27)	
		Presence	3826 (12.73)	
	Self-rated health status (score of 0-100), median (IQR)			79.00 (63.00-87.00)
	Health literacy (score of 4-16), median (IQR)			10.00 (8.00-12.00)
	eHealth literacy (score of 10-20), median (IQR)			18.00 (15.00-20.00)
**Interpersonal network level**				
	**Marital status, n (%)**			
		No spouse	8900 (29.61)	
		Married	21,154 (70.39)	
	Family health (score of 10-50), median (IQR)			40.00 (36.00-45.00)
**Community level**				
	**Career status, n (%)**			
		Student	4681 (15.58)	
		Employed	18,345 (61.04)	
		Unemployed	7028 (23.38)	
	**Urban-rural distribution, n (%)**			
		Rural	9319 (31.01)	
		Urban	20,735 (68.99)	
	**Number of house properties, n (%)**			
		0	3069 (10.21)	
		1	17,220 (57.3)	
		≥2	9765 (32.49)	
	**Average monthly household income, n (%)**			
		≤¥3000 (US $415.94)	8781 (29.22)	
		¥3001 to ¥6000 (US $416.08 to $831.88)	13,428 (44.68)	
		≥¥6001 (US $832.02)	7845 (26.1)	
	Perceived social support (score of 3-21), median (IQR)			16.00 (13.00-18.00)
**Policy level**				
	**Medical insurance type, n (%)**			
		No insurance coverage	1497 (4.98)	
		Resident basic medical insurance	17,920 (59.63)	
		Employee basic medical insurance	7754 (25.8)	
		Commercial insurance	2883 (9.59)	
	Level of trust in health information robots (score of 0-100), median (IQR)			60.00 (44.00-77.00)

^a^Percentages might not add up to 100% due to rounding.

### Factors Associated With the Level of Trust in Health Information Robots

The results of the univariate generalized linear model analysis indicated that most variables are associated with the level of trust in robots providing health information (Table 2). In addition, the results of the multivariate generalized linear model analysis revealed that the following characteristics were positively associated with participants’ level of trust in health information robots compared to individuals without these characteristics: those diagnosed with chronic diseases (β=2.45, 95% CI 1.78-3.13); those who exhibited the personality traits of agreeableness (β=0.43, 95% CI 0.22-0.64) and openness (β=0.42, 95% CI 0.24-0.61); those who had an educational level of junior college or higher (β=0.93, 95% CI 0.03-1.83); and those who reported higher self-rated health status (β=0.16, 95% CI 0.14-0.18), health literacy (β=0.12, 95% CI 0.02-0.22), anxiety symptoms (β=1.96, 95% CI 0.90-3.03), family health (β=0.08, 95% CI 0.02-0.13), number of house properties (1: β=1.87, 95% CI 0.94-2.81; ≥2: β=2.22, 95% CI 1.21-3.22), average monthly household income (¥3001 to ¥6000 [US $416.08 to $831.88]: β=1.79, 95% CI 1.13-2.45; ≥¥6001 [US $832.02]: β=2.86, 95% CI 2.05-3.67), perceived social support (β=0.46, 95% CI 0.38-0.54), and participants had a higher medical insurance coverage (β=1.77, 95% CI 0.26-3.27). Negative associations were observed for older age (β=−0.03, 95% CI –0.06 to –0.01), neuroticism (β=−0.25, 95% CI –0.45 to –0.06), and urban residence (β=−1.10, 95% CI −1.73 to −0.46; Table 3).

**Table 2:** Univariate generalized linear model analysis of associations between study variables and the level of trust in health information robots (N=30,054)^a^.

Variable				β^b^ (95% CI)		*P* value
**Individual characteristic level**						
	Age			−0.09 (−0.11 to –0.07)		<.001
	**Sex (reference: male)**					
		Female	−0.11 (−0.66 to 0.43)		.68	
	**Educational level (reference: primary school and lower)**					
		Junior high school and senior school	1.20 (0.52 to 1.87)		.001	
		Junior college and higher	3.60 (2.93 to 4.27)		<.001	
	**Diagnosed with a chronic disease (reference: no)**					
		Yes	−0.81 (−1.39 to –0.23)		.007	
	Extraversion			0.63 (0.47 to 0.80)		<.001
	Agreeableness			1.07 (0.88 to 1.26)		<.001
	Conscientiousness			0.58 (0.41 to 0.75)		<.001
	Neuroticism			0.34 (0.15 to 0.53)		<.001
	Openness			1.00 (0.82 to 1.17)		<.001
	Health-related quality of life			11.96 (9.73 to 14.18)		<.001
	Concern regarding privacy breaches in artificial intelligence health-monitoring devices			0.01 (−0.001 to 0.02)		.09
**Individual behavior level**						
	**Depression symptoms (reference: absence)**					
		Presence	−2.04 (−2.73 to –1.35)		<.001	
	**Anxiety symptoms (reference: absence)**					
		Presence	−0.92 (−1.74 to –0.10)		.03	
	Self-rated health status			0.20 (0.19 to 0.22)		<.001
	Health literacy			0.12 (0.02 to 0.22)		.02
	eHealth literacy			0.01 (−0.05 to 0.06)		.75
**Interpersonal network level**						
	**Marital status (reference: no spouse)**					
		Married	−2.1 7 (−2.77 to –1.57)		<.001	
	Family health			0.45 (0.40 to 0.49)		<.001
**Community level**						
	**Career status (reference: student)**					
		Employed	−2.30 (−3.08 to –1.53)		<.001	
		Unemployed	−4.83 (−5.72 to –3.94)		<.001	
	**Urban-rural distribution (reference: rural)**					
		Urban	0.83 (0.24 to 1.42)		.006	
	**Number of house properties (reference: 0)**					
		1	2.99 (2.07 to 3.92)		<.001	
		≥2	4.60 (3.62 to 5.58)		<.001	
	**Average monthly household income (reference: ≤¥3000 [US $415.94])**					
		¥3001 to ¥6000 (US $416.08 to $831.88)	2.81 (2.16 to 3.45)		<.001	
		≥¥6001 (US $832.02)	4.95 (4.22 to 5.68)		<.001	
	Perceived social support			0.77 (0.70 to 0.84)		<.001
**Policy level**						
	**Medical insurance type (reference: out of pocket)**					
		Resident basic medical insurance	1.48 (0.21 to 2.75)		.02	
		Employee basic medical insurance	1.44 (0.10 to 2.77)		.04	
		Commercial insurance	3.90 (2.39 to 5.41)		<.001	

^a^All reference variables take the value of 0.

^b^Regression coefficient.

**Table 3:** Multivariate generalized linear model analysis of associations between study variables and the level of trust in health information robots (N=30,054)^a^.

Variable			β^b^ (95% CI)	*P* value
**Individual characteristic level**				
	Age		−0.03 (−0.06 to –0.01)	.02
	**Educational level (reference: primary school and lower)**			
		Junior high school and senior school	−0.25 (−1.03 to 0.52)	.52
		Junior college and higher	0.93 (0.03 to 1.83)	.04
	**Diagnosed with a chronic disease (reference: no)**			
		Yes	2.45 (1.78 to 3.13)	<.001
	Extraversion		0.15 (−0.02 to 0.32)	.09
	Agreeableness		0.43 (0.22 to 0.64)	<.001
	Conscientiousness		0.02 (−0.17 to 0.21)	.84
	Neuroticism		−0.25 (−0.45 to –0.06)	.01
	Openness		0.42 (0.24 to 0.61)	<.001
	Health-related quality of life		2.11 (−0.34 to 4.57)	.09
**Individual behavior level**				
	**Depression symptoms (reference: absence)**			
		Presence	−0.03 (−0.95 to 0.89)	.95
	**Anxiety symptoms (reference: absence)**			
		Presence	1.96 (0.90 to 3.03)	<.001
	Self-rated health status		0.16 (0.14 to 0.18)	<.001
	Health literacy		0.12 (0.02 to 0.22)	.02
**Interpersonal network level**				
	**Marital status (reference: no spouse)**			
		Married	−0.77 (−1.67 to 0.12)	.09
	Family health		0.08 (0.02 to 0.13)	.006
**Community level**				
	**Career status (reference: student)**			
		Employed	−0.57 (−1.59 to 0.45)	.27
		Unemployed	−1.28 (−2.57 to 0.003)	.05
	**Urban-rural distribution (reference: rural)**			
		Urban	−1.10 (−1.73 to –0.46)	.001
	**Number of house properties (reference: 0)**			
		1	1.87 (0.94 to 2.81)	<.001
		≥2	2.22 (1.21 to 3.22)	<.001
	**Average monthly household income (reference: ≤¥3000 [US $415.94])**			
		¥3001 to ¥6000 (US $416.08 to $831.88)	1.79 (1.13 to 2.45)	<.001
		≥¥6001 (US $832.02)	2.86 (2.05 to 3.67)	<.001
	Perceived social support		0.46 (0.38 to 0.54)	<.001
**Policy level**				
	**Medical insurance type (reference: out of pocket)**			
		Resident basic medical insurance	0.65 (−0.61 to 1.91)	.31
		Employee basic medical insurance	−0.57 (−1.94 to 0.80)	.41
		Commercial insurance	1.77 (0.26 to 3.27)	.02

^a^All reference variables take the value of 0.

^b^Regression coefficient.

### Graphical LASSO Network

**Figure 2:**
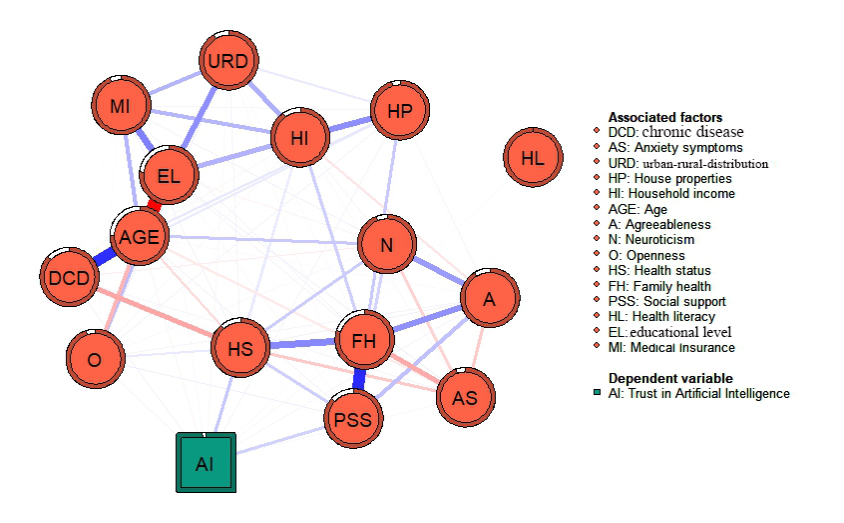
Network structure of the level of trust in health information robots and its associated factors. A: agreeableness; AI: level of trust in robots; AS: anxiety symptoms; DCD: chronic disease; EL: educational level; FH: family health; HI: household income; HL: health literacy; HP: house properties; HS: health status; MI: medical insurance; N: neuroticism; O: openness; PSS: social support; URD: urban-rural distribution.

**Figure 3:**
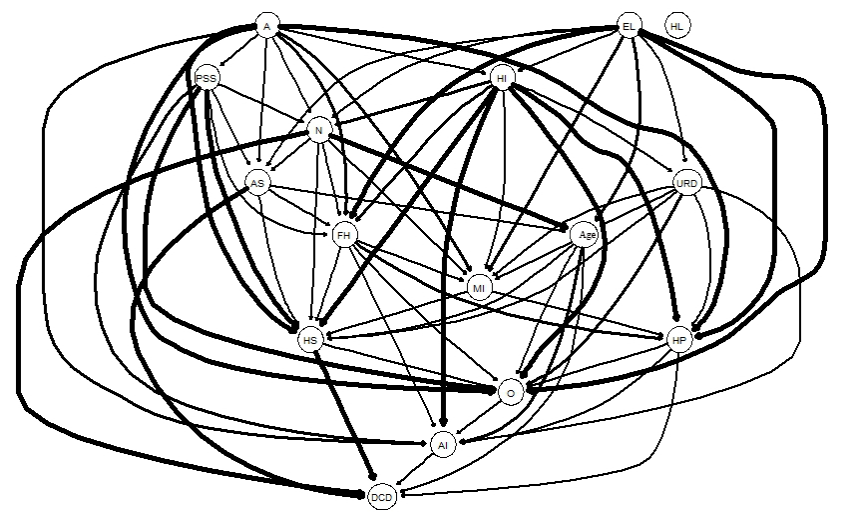
Directed acyclic graph—the thickness of the arrows denotes their importance to the overall network model. A: agreeableness; AI: level of trust in robots; AS: anxiety symptoms; DCD: chronic disease; EL: educational level; FH: family health; HI: household income; HL: health literacy; HP: house properties; HS: health status; MI: medical insurance; N: neuroticism; O: openness; PSS: social support; URD: urban-rural distribution.

A description of the participants’ trust levels in health information robots and the significant factors derived from the multivariate generalized linear model are presented in Table 4. Figures 2 and 3 show a visual network illustrating the interconnections between the level of trust in health information robots and the significantly associated factors derived from the multivariate generalized linear model. Table 4 and Figure 2 further illustrate that family health had the highest expected influence score among the nodes in the network regarding the level of trust in health information robots and its associated factors. This was followed by perceived social support. The partial correlation coefficients are listed in Table S1 in Multimedia Appendix 1.

Figure S2 in Multimedia Appendix 1 demonstrates the stability of the network using the case-dropping bootstrap method, which was conducted 1000 times. The CS-Cs for the expected influence were found to be 0.75, suggesting that the network structure would remain intact even if 75% of the sample data were excluded. Centrality measures derived from the network established in this study indicated a high level of stability. The results from the nonparametric bootstrap procedure revealed that most comparisons concerning edge weights and expected node influence were statistically significant (Figures S3-S5 in Multimedia Appendix 1). In addition, the narrow 95% CIs obtained from the bootstrapping process emphasize the reliability of the network edges (Figure S6 in Multimedia Appendix 1).

**Table 4:** Descriptive statistics of the level of trust in health information robots and the significantly associated factors derived from the multivariate generalized linear model for the participants (N=30,054).

Item abbreviation	Item content	Node expected influence^a^	Predictability^a^
AI	The level of trust in robots	0.304	0.041
DCD	Chronic disease	0.224	0.245
AS	Anxiety symptoms	−0.372	0.105
URD	Urban-rural distribution	0.519	0.161
HP	House properties	0.467	0.088
HI	Household income	0.736	0.187
AGE	Age	−0.050	0.434
A	Agreeableness	0.396	0.166
N	Neuroticism	0.360	0.115
O	Openness	0.202	0.104
HS	Health status	0.284	0.217
FH	Family health	0,922	0.358
PSS	Social support	0.747	0.248
HL	Health literacy	0.006	0
EL	Educational level	0.276	0.413
MI	Medical insurance	0.703	0.123

^a^The values of node expected influence and predictability were raw data from the network.

### Directed Acyclic Graph

**Figure 4:**
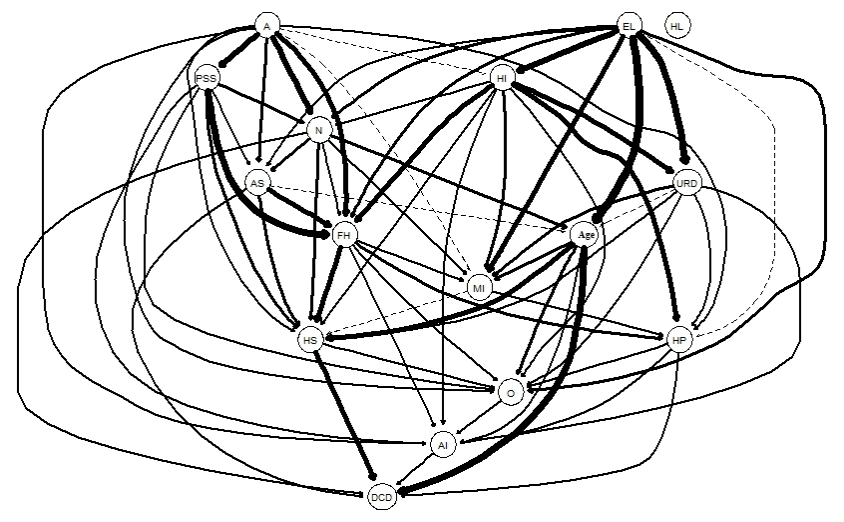
Directed acyclic graph—arrow thickness indicates directional probability. A: agreeableness; AI: level of trust in robots; AS: anxiety symptoms; DCD: chronic disease; EL: educational level; FH: family health; HI: household income; HL: health literacy; HP: house properties; HS: health status; MI: medical insurance; N: neuroticism; O: openness; PSS: social support; URD: urban-rural distribution.

Figure 3 illustrates the significance of each edge within the overall directed acyclic graph framework (the thickness of the edges reflects the alteration in the BIC resulting from the removal of the edge from the directed acyclic graph). A greater edge thickness signifies the edge’s increased importance for the model fit. When the level of trust in health information robots was considered as the dependent variable, the most significant independent variable in the directed acyclic graph was perceived social support (alteration in BIC value: −80.21). Table S2 in Multimedia Appendix 1 shows the changes in the BIC values for all edges. Figure 4 shows the directional probabilities associated with each edge. A greater edge thickness implies that the current direction was found in a greater fraction of the bootstrapped directed acyclic graphs. The thickest edge connected neuroticism to whether a chronic disease had been diagnosed (0.856; ie, this edge was oriented in that direction in 856/1000, 85.6% of the bootstrapped directed acyclic graphs). The specific directional probabilities of all edges in Figure 4 are listed in Table S2 in Multimedia Appendix 1. Structurally, agreeableness and educational level appeared upstream of the entire directed acyclic graph, directly influencing the dependent and independent variables.

## Discussion

### Principal Findings

This study assesses the level of trust in health information robots and the associated factors within the Chinese population using a nationally representative sample. From a socioecological model perspective, this study identified several factors associated with the level of trust in health information robots. At the individual characteristic level, these factors include older age; the presence of diagnosed chronic diseases; educational level; and personality traits such as agreeableness, neuroticism, and openness. At the individual behavior level, factors included higher levels of anxiety symptoms, self-rated health status, and health literacy. At the interpersonal network level, greater family health was relevant. At the community level, urban-rural distribution, number of house properties, average monthly household income, and perceived social support were relevant. At the policy level, the type of medical insurance was a significant factor. In addition, using a network approach, central indicators were identified in the network of the level of trust in health information robots and its associated factors, including family health and perceived social support. Agreeableness and educational level appeared upstream of the entire directed acyclic graph, directly influencing the level of trust in health information robots.

This study demonstrated that the socioecological model can effectively clarify the factors associated with the level of trust in health information robots from a relatively broad perspective. Specifically, by applying a socioecological model, the results revealed that the factors associated with the level of trust in health information robots can be found predominantly at the individual characteristic level. Relevant factors were also identified at the levels of individual behavior, interpersonal networks, community, and policy. Previous studies that conducted cross-sectional surveys identified self-efficacy and empirical knowledge as factors influencing trust in public health information during the COVID-19 pandemic [66,67]. One literature review identified 19 factors that were positively associated with trust in and credibility of online health information, whereas 11 factors were negatively related [23]. Another systematic review highlighted that adolescents’ perceptions of trust in health information shared on social media were related to 3 key aspects: other users, content, and social media platforms [68]. These studies emphasize the importance of exploring the factors that influence the level of trust in health information. On the basis of the socioecological model and network approach, our study offers a novel perspective on the factors associated with the level of trust in health information robots. Future in-depth analyses and targeted intervention strategies aimed at enhancing trust in health information robots should consider these perspectives.

Our study demonstrated a significant negative association between higher educational levels and trust in health information robots. In addition, the educational level was upstream in the entire directed acyclic graph, directly influencing the level of trust in health information robots. This may be because of 2 reasons. First, individuals with higher education generally have more opportunities to access health-related resources, making them less reliant on the health information provided by robots [69]. Second, individuals with higher educational levels have a greater ability to discover, understand, and evaluate health-related information, which enables them to better access and analyze the sources and reliability of that information [70]. However, this may also lead to doubts regarding the accuracy and applicability of the health information provided by robots, particularly for complex health issues. Therefore, the understanding and application of AI technology should be incorporated into educational content, enabling learners to gain a deeper understanding of the working principles, algorithms, and limitations of robots so that they can more comprehensively analyze and evaluate the scientific validity and effectiveness of the health information provided by them.

We also observed that individuals with higher scores on the agreeableness dimension of the BFI-10 tended to place greater trust in health information robots. In addition, agreeableness was positioned upstream in the entire directed acyclic graph, directly influencing the level of trust in robots that deliver health information. Agreeable individuals often have a better understanding of others’ emotions and behaviors, which gives them an advantage when analyzing robot performance in specific situations [71]. This emotional awareness enables them to accept the health information provided by the robots more readily, thereby demonstrating a higher level of trust. Our study suggests that personalized designs should be implemented in the development of health information robots considering users’ personality traits to enhance trust in the information provided by these robots.

Within the framework of centrality indicators, family health was identified as having the most significant influence. Family health is defined as a resource at the family unit level that emerges from the interplay between each family member’s health, interactions, and abilities and the family’s physical, emotional, economic, social, and medical resources [72]. On the basis of this characteristic, one possible explanation is that family health helps mitigate various physical and mental issues, including depression and anxiety, by fostering stronger relationships and more effective family dynamics, thereby maintaining individuals in a positive state [73]. When individuals are in a positive emotional state, they are better able to rationally evaluate information sources, thereby increasing their level of trust in health information robots [74]. Another possible reason is that family health can indirectly promote physical health by enhancing the prevention and management of chronic diseases [75]. Individuals who have achieved positive health outcomes by using health information in the past are more likely to be open to new health recommendations even if these suggestions come from digital information [76]. Therefore, initiatives focused on enhancing family health may indirectly help prevent and manage the level of trust in health information robots at the population level.

In this study, the second most significant central node indicator was perceived social support, which was positively associated with the level of trust in health information robots. This result aligns with those of a previous study that found that social support directly influenced online health information–seeking behaviors among 451 older adults diagnosed with coronary heart disease [77]. One possible explanation is that high levels of social support result in emotional care and understanding, which helps individuals mitigate mental health problems [78]. A reduction in psychological issues is often accompanied by a stronger coping ability [79], allowing individuals to better process the health information provided by robots, thereby enhancing their level of trust in them [73]. Health information robots constitute a form of social support. Consequently, individuals with heightened recognition and perception of social support are more inclined to accept the diverse types of social support provided by robots. Therefore, recognizing these 2 central indicators is crucial for developing intervention strategies to enhance trust in health information robots.

Our study indicated a significant negative association between participants’ increasing age and their level of trust in health information robots. A possible explanation is that, with increasing age, individuals are more likely to place trust in established sources with a history of reliability as opposed to embracing novel robotic technologies that are less familiar to them [80]. Our study extends those of a previous study that indicate that older adults exhibit lower trust in health information provided by the internet; however, the aforementioned study did not explicitly explore the trust of older adults in health information provided by robots [81]. However, as a form of AI technology, robots are typically connected to reliable medical databases, enabling them to provide accurate and trustworthy health information, thereby compensating for the shortcomings of older adults in their information retrieval and judgment abilities [82]. Therefore, increasing older adults’ trust in the health information provided by robots helps meet their need for professional medical advice, avoids exposure to inaccurate or misleading health information, and more effectively assists older adults in making informed health decisions. Simultaneously, this may ensure equitable access to requisite medical assistance and health management for the entire older adult population in the future.

Our study found that, compared to individuals without chronic diseases, those with chronic diseases exhibited a higher level of trust in health information robots. The health information provided by medical robots is more accurate and reliable in certain health care institutions and chronic disease outpatient clinics in China [83]. Patients with chronic diseases typically require continuous health management and information support [84]. Therefore, compared with individuals without chronic diseases, those with chronic diseases are more inclined to trust the health information provided by robots to obtain reliable and effective support for managing their health conditions. Our study supports a previous study that found that, when evaluating the internet as a channel for obtaining health information, individuals in poor health were more likely to seek health information online than those in good health [85]. However, our study focused on the health information provided by robots. Compared with traditional internet searches, robots can engage in more personalized and interactive communication with users through natural language processing technology, thus reducing information overload and making it easier for patients with chronic diseases to find information relevant to their health conditions [86]. Our study suggests that health care institutions should develop robots that can tailor health information to the unique health needs of individuals, thereby meeting the information needs of patients with chronic diseases. Through the health information provided using these robots, patients can better understand and manage their health conditions, thereby improving their quality of life.

We also observed that individuals with higher scores on the openness dimension of the BFI-10 tended to place greater trust in health information robots. In contrast, participants who scored higher on neuroticism showed lower levels of trust in health information robots. One possible explanation for openness is that, compared to other personality types, people with higher levels of openness possess greater cognitive abilities and are more likely to engage in social and technological environments, which may make them more willing to trust health information provided by robots [87]. Another potential explanation for neuroticism is that, as a stable personality trait, it corresponds to and predisposes individuals to experience adverse emotional states [88]. Under the influence of negative emotions, people may be more inclined to adopt emotion-focused coping strategies, which could lead them to make inappropriate decisions, resulting in decreased trust in the health information provided by robots [89]. Although our study did not directly reveal an association between concerns about privacy breaches and the level of trust in health information provided by robots, a previous study indicated that individuals with higher levels of neuroticism are often more prone to privacy concerns, which may hinder their willingness to authorize the use of personal information when using applications [90]. This highlights the importance of considering personality traits and privacy issues when enhancing trust in health information robots. Future studies should explore the interactions between privacy issues and personality factors to enhance user trust in robots that provide health-related information.

Our study indicated that individuals exhibiting higher levels of anxiety symptoms were more inclined to trust health information robots. One possible reason is that individuals with anxiety symptoms tend to have a heightened focus on and concern about their health issues, which drives them to actively seek information to alleviate their internal distress [91]. Consequently, for individuals with anxiety, robots may be perceived as a quick and convenient resource, enhancing trust in the information offered by these robots. This is consistent with the hypochondriasis theory, which suggests that excessive preoccupation with one’s health constitutes distorted cognition that may lead individuals to make judgments about illness and other matters that differ from those of others [92]. Therefore, in addition to examining the impact of psychological factors on individuals’ trust in the health information provided by robots, it is crucial to enhance the public’s understanding and recognition of health information sources. This will help individuals make more informed decisions when accessing health information provided by robots and enhance their trust in these information sources.

Our findings revealed that individuals with higher levels of self-rated health status were more inclined to trust robots that provide them with health information. This aligns with a previous study indicating that individuals in better health are more closely linked to the information they find online [93]. One possible explanation is that self-reported health status is influenced not only by an individual’s physical health but also by factors related to satisfaction and psychological well-being [94,95]. Higher levels of satisfaction and excellent psychological status are often associated with positive emotions, which make individuals more willing to accept and trust health information [74]. Our study suggests that health information disseminators can use robots to provide health education information when individuals with better self-rated health optimize human-robot interactions, which is crucial for advancing the application of AI in the medical and health fields.

Our study found that individuals with higher levels of health literacy were more likely to trust health information robots. A previous study involving 4974 American adults revealed that individuals with poor health literacy were less inclined to use health IT tools or consider them accessible or useful [96]. However, our study validates this result across different sociocultural contexts and for diverse populations. A possible reason for this is the characteristics of health literacy. Health literacy quality encompasses the manner in which individuals gather, understand, apply, and communicate health information to make educated decisions [97]. Individuals with high health literacy typically possess stronger information evaluation skills, enabling them to better understand and analyze the sources and content of their health information [98]. Consequently, those with high health literacy are more inclined to trust the health information provided by robots because they can correctly interpret and apply such information. Although our study did not find an association between eHealth literacy and the level of trust in the health information provided by robots, previous research has indicated that users with higher eHealth literacy are more capable of using mobile health apps to access the health information they require [99]. A possible reason is that, although both robots and mobile health devices are applications of AI, individuals may still hold reservations about the capabilities and accuracy of robots in specific contexts, which may lead them to be more inclined to trust the information provided by mobile health devices [100]. Therefore, future studies should investigate temporal changes in eHealth literacy and their effects on users’ trust in the health information provided by robots to provide a comprehensive understanding of user trust in robotic health information. In addition, establishing effective collaborative mechanisms among various medical and health service institutions can enhance public health literacy, ensuring that individuals are better equipped to evaluate and apply the health information they receive.

Our study found that, compared to individuals living in rural areas, those residing in urban areas exhibited lower levels of trust in health information robots. Urban residents often experience higher levels of stress than their rural counterparts owing to factors such as housing instability and limited job opportunities, making them more cautious about adopting new technologies [101]. Therefore, despite the positive impact of the robust economic development in urban areas, individuals may still be more inclined to rely on traditional and familiar sources of health information [102]. This may lead to a decreased level of trust in the health information provided by robots. Designers can customize robots to provide personalized health information services based on the needs of residents. Policy makers should leverage community resources to strengthen connections between rural and urban areas to narrow the urban-rural divide, thereby facilitating residents’ acceptance of the health information provided by robots.

Our study found that the number of properties owned and the average monthly household income were positively associated with individuals’ trust in health information robots. Previous studies support our findings, indicating that socioeconomic characteristics such as individual income and economic freedom are positively associated with trust in public health information and interpersonal trust [103,104]. However, in the context of the increasing prevalence of AI technologies, our study explored the association between household economic status and the level of trust in health information robots. One possible reason is that individuals with better economic conditions typically have a stronger need for information [105], making them more willing to actively seek and obtain health information through various channels [106]. Consequently, individuals with better economic conditions are more likely to accept robots as a new source of health information, leading to a higher level of trust in this new source. Policy makers should develop targeted strategies for groups at different economic levels and provide more education and training to enhance trust in health information robots.

Our study found that, at the policy level, having a reduced out-of-pocket insurance cost was significantly positively associated with the level of trust in health information robots. A possible reason is that individuals with commercial insurance often have a higher socioeconomic status and may be more trusting of the health information provided by robots because they have access to more resources and information channels to better understand the latest developments in robotics [107]. However, the interpretation of these results requires careful consideration of several factors. First, most participants used commercial insurance and socialized medicine significantly less than other forms of medical insurance. Second, in China, individuals with commercial insurance and socialized medicine frequently have additional types of insurance, such as basic resident medical insurance, which may result in an overlap among commercial insurance, socialized medicine, and other medical insurance forms. Therefore, the use figures for commercial insurance and socialized medicine may not fully represent these categories as distinct entities. Although the number of individuals who use commercial insurance is relatively low, their trust in health information from robots boosts national social and medical security.

Our study is based on the behavioral intention component of the TAM, examining research perspectives, and selecting variables according to a socioecological model. We explored the factors associated with the level of trust in health information robots from multiple dimensions, revealing causal associations and central indicators among these factors. However, additional associated factors must be considered to further investigate whether individuals can accept this new technology to receive health information from robots. For instance, a previous systematic review indicated that, in addition to trust as an emotional attitude associated with people’s acceptance of robots, an individual’s previous direct or indirect contact with robots is also associated with their level of acceptance [21]. Therefore, future studies should further explore individuals’ acceptance of health information robots based on the additional dimensions of the TAM. For instance, concerns regarding potential misinformation, inconsistencies, or verification issues related to health information provided by robots may arise in the future. In addition, the perceived usefulness of the health information delivered by robots and their ability to provide personalized health information could be associated with individuals’ acceptance of these robots.

This study had some limitations. First, this study used self-reported data to evaluate the level of trust in health information robots and its associated factors. This approach could introduce biases owing to memory issues and the desire to provide socially acceptable responses. Future research should incorporate more objective assessment techniques to confirm these results and enhance the accuracy of the findings. Second, although this study used a nationally representative sample, only data from China were included in the analysis. Therefore, it is necessary to validate our findings in other countries. Finally, although this study identified several factors associated with individuals’ trust in health information robots based on the TAM and socioecological model, it is essential to consider other associated factors to further verify whether individuals can accept receiving health information from robots. For example, the accuracy and reliability of the health information provided by robots, as well as the ability of robots to tailor information to individuals’ unique health needs, should be considered. Therefore, future studies should include a comprehensive assessment of other factors and elucidate their complex interplay to gain a more thorough understanding of the factors affecting people’s trust in health information robots.

### Conclusions

This study identified key factors associated with the level of trust in health information robots within a multitiered socioecological model framework. Agreeableness and educational level appeared upstream of the entire directed acyclic graph, directly influencing the level of trust in health information robots. Finally, central indicators were identified in the network regarding the level of trust in health information robots and its associated factors, including family health and perceived social support. These results provide a new perspective for the application and development of AI IT. Future research can leverage these associated factors to enhance individuals’ acceptance of and compliance with health information. Furthermore, by enhancing public trust in the health information provided by robots, it is possible to bridge the gap in access to health information among individuals from diverse socioeconomic backgrounds, thereby improving the efficiency and equity of medical services.

## Appendix

Multimedia Appendix 1 Correlation matrix.

## Abbreviations

AI: artificial intelligence
BFI-10: Big Five Inventory–10
BIC: Bayesian information criterion
CS-C: correlation stability coefficient
eHEALS-SF: eHealth Literacy Scale–Short Form.
FHS-SF: Family Health Scale–Short Form
GAD-7: Generalized Anxiety Disorder–7
HLS-SF-4: Health Literacy Scale–Short Form
LASSO: least absolute shrinkage and selection operator
PHQ-9: Patient Health Questionnaire–9
PSSS: Perceived Social Support Scale
TAM: technology acceptance model
