# Supplementary material for: Factors Associated With the Level of Trust in Health Information Robots Among the General Population From a Socioecological Model Perspective: Network Analysis
Source: J Med Internet Res. 2025 Jun 13;27:e68299. doi: 10.2196/68299 (PMC12205264; doi:10.2196/68299)
Supplement: Multimedia Appendix 1 [file jmir_v27i1e68299_app1.docx]

Table S1. Correlation matrix of the level of trust in robots providing health information and the significant associated factors

| Items | AI | DCD | AS | URD | HP | HI | AGE | A | N | O | HS | FH | PSS | HL | EL | MI |
| --- | --- | --- | --- | --- | --- | --- | --- | --- | --- | --- | --- | --- | --- | --- | --- | --- |
| AI |  | 0.02 | 0.01 | -0.01 | 0.02 | 0.04 | -0.02 | 0.02 | 0.00 | 0.03 | 0.10 | 0.01 | 0.08 | 0.01 | 0.01 | 0.00 |
| DCD | 0.02 |  | 0.02 | 0.00 | 0.03 | -0.01 | 0.36 | 0.00 | -0.03 | 0.00 | -0.15 | 0.00 | 0.00 | 0.00 | -0.01 | 0.00 |
| AS | 0.01 | 0.02 |  | 0.00 | 0.00 | 0.01 | -0.04 | -0.09 | -0.07 | 0.00 | -0.09 | -0.15 | 0.00 | 0.00 | 0.03 | 0.00 |
| URD | -0.01 | 0.00 | 0.00 |  | 0.04 | 0.14 | -0.02 | 0.00 | 0.00 | 0.03 | 0.02 | 0.01 | -0.01 | 0.00 | 0.20 | 0.13 |
| HP | 0.02 | 0.03 | 0.00 | 0.04 |  | 0.19 | 0.04 | -0.02 | 0.00 | 0.02 | 0.00 | 0.09 | 0.00 | 0.00 | 0.03 | 0.02 |
| HI | 0.04 | -0.01 | 0.01 | 0.14 | 0.19 |  | 0.00 | -0.04 | 0.02 | 0.01 | 0.04 | 0.09 | 0.00 | 0.00 | 0.13 | 0.12 |
| AGE | -0.02 | 0.36 | -0.04 | -0.02 | 0.04 | 0.00 |  | 0.01 | 0.09 | -0.13 | -0.07 | 0.00 | 0.02 | 0.00 | -0.44 | 0.13 |
| A | 0.02 | 0.00 | -0.09 | 0.00 | -0.02 | -0.04 | 0.01 |  | 0.17 | 0.02 | 0.03 | 0.19 | 0.11 | 0.00 | 0.00 | 0.01 |
| N | 0.00 | -0.03 | -0.07 | 0.00 | 0.00 | 0.02 | 0.09 | 0.17 |  | 0.01 | 0.09 | 0.01 | 0.08 | 0.00 | -0.02 | 0.02 |
| O | 0.03 | 0.00 | 0.00 | 0.03 | 0.02 | 0.01 | -0.13 | 0.02 | 0.01 |  | 0.02 | 0.04 | 0.03 | 0.00 | 0.12 | 0.00 |
| HS | 0.10 | -0.15 | -0.09 | 0.02 | 0.00 | 0.04 | -0.07 | 0.03 | 0.09 | 0.02 |  | 0.21 | 0.08 | 0.00 | 0.00 | 0.02 |
| FH | 0.01 | 0.00 | -0.15 | 0.01 | 0.09 | 0.09 | 0.00 | 0.19 | 0.01 | 0.04 | 0.21 |  | 0.37 | 0.00 | 0.02 | 0.02 |
| PSS | 0.08 | 0.00 | 0.00 | -0.01 | 0.00 | 0.00 | 0.02 | 0.11 | 0.08 | 0.03 | 0.08 | 0.37 |  | 0.00 | -0.01 | 0.00 |
| HL | 0.01 | 0.00 | 0.00 | 0.00 | 0.00 | 0.00 | 0.00 | 0.00 | 0.00 | 0.00 | 0.00 | 0.00 | 0.00 |  | 0.00 | 0.00 |
| EL | 0.01 | -0.01 | 0.03 | 0.20 | 0.03 | 0.13 | -0.44 | 0.00 | -0.02 | 0.12 | 0.00 | 0.02 | -0.01 | 0.00 |  | 0.22 |
| MI | 0.00 | 0.00 | 0.00 | 0.13 | 0.02 | 0.12 | 0.13 | 0.01 | 0.02 | 0.00 | 0.02 | 0.02 | 0.00 | 0.00 | 0.22 |  |

Notes: A: agreeableness; AI: level of trust in robots; AS: anxiety symptoms; DCD: chronic disease; EL: educational level; FH: family health; HI: household income; HL: health literacy; HP: house properties; HS: health status; MI: medical insurance; N: neuroticism; O: openness; PSS: social support; URD: urban-rural distribution.

Table S2. Arrows weight values in the directed acyclic graphs

| Arrow | | Value determining arrow thickness | |
| --- | --- | --- | --- |
| From | To | BIC | Directional probability |
| AI | DCD | -16.18 | 0.51 |
| AS | DCD | -5.21 | 0.76 |
| AS | AGE | 0.09 | 0.57 |
| AS | HS | -211.95 | 0.63 |
| AS | FH | -591.79 | 0.59 |
| URD | AI | -0.18 | 0.52 |
| URD | HP | -15.04 | 0.63 |
| URD | AGE | 1.67 | 0.68 |
| URD | O | -11.72 | 0.57 |
| URD | HS | -2.49 | 0.59 |
| URD | MI | -204.00 | 0.53 |
| HP | AI | -5.04 | 0.56 |
| HP | DCD | -11.73 | 0.59 |
| HP | O | -9.38 | 0.59 |
| HI | AI | -27.84 | 0.79 |
| HI | URD | -482.05 | 0.63 |
| HI | HP | -582.98 | 0.82 |
| HI | N | -31.03 | 0.69 |
| HI | O | -0.72 | 0.77 |
| HI | HS | -36.17 | 0.81 |
| HI | FH | -315.52 | 0.71 |
| HI | MI | -281.54 | 0.65 |
| AGE | AI | -26.09 | 0.69 |
| AGE | DCD | -3415.51 | 0.59 |
| AGE | O | -274.33 | 0.55 |
| AGE | HS | -421.15 | 0.57 |
| AGE | MI | -229.03 | 0.52 |
| A | AI | -3.62 | 0.74 |
| A | AS | -283.36 | 0.64 |
| A | HP | -3.47 | 0.86 |
| A | HI | 5.13 | 0.56 |
| A | N | -810.95 | 0.65 |
| A | O | -12.42 | 0.81 |
| A | HS | -9.60 | 0.76 |
| A | FH | -560.13 | 0.69 |
| A | PSS | -1002.56 | 0.63 |
| A | MI | 1.35 | 0.71 |
| N | DCD | -28.80 | 0.86 |
| N | AS | -197.95 | 0.54 |
| N | AGE | -88.40 | 0.75 |
| N | HS | -145.51 | 0.61 |
| N | FH | -12.21 | 0.54 |
| N | MI | -9.09 | 0.56 |
| O | AI | -11.09 | 0.51 |
| HS | DCD | -326.92 | 0.83 |
| HS | O | -2.35 | 0.51 |
| FH | AI | -19.85 | 0.52 |
| FH | HP | -210.23 | 0.69 |
| FH | O | -12.62 | 0.62 |
| FH | HS | -753.03 | 0.51 |
| FH | MI | -3.88 | 0.51 |
| PSS | AI | -80.21 | 0.71 |
| PSS | AS | -29.74 | 0.62 |
| PSS | N | -161.85 | 0.52 |
| PSS | O | -9.95 | 0.78 |
| PSS | HS | -108.86 | 0.78 |
| PSS | FH | -2800.40 | 0.66 |
| EL | AS | -15.72 | 0.61 |
| EL | URD | -1311.75 | 0.56 |
| EL | HP | 5.15 | 0.82 |
| EL | HI | -1276.62 | 0.51 |
| EL | AGE | -4474.68 | 0.72 |
| EL | N | -83.35 | 0.64 |
| EL | O | -251.71 | 0.80 |
| EL | FH | -90.57 | 0.75 |
| EL | MI | -651.65 | 0.66 |
| MI | HP | -13.99 | 0.53 |
| MI | HS | 0.64 | 0.53 |

Notes: BIC: Change in Bayesian Information Criterion when the arrow is removed from the network. BIC values determine arrow thickness in Figure 8 (reflecting the importance of that edge to the network structure). For the BIC values, negative values correspond to decreases in the network score caused by the removal of the arrow. In other words, negative scores indicate that model fit improves with the presence of that arrow. Directional probability values determine arrow thickness in Figure 9 (reflecting the frequency with which the arrow was present in that direction in the 1000 bootstrapped networks). A: agreeableness; AI: level of trust in robots; AS: anxiety symptoms; DCD: chronic disease; EL: educational level; FH: family health; HI: household income; HL: health literacy; HP: house properties; HS: health status; MI: medical insurance; N: neuroticism; O: openness; PSS: social support; URD: urban-rural distribution.
